# Procyanidin B2 Protects Neurons from Oxidative, Nitrosative, and Excitotoxic Stress

**DOI:** 10.3390/antiox6040077

**Published:** 2017-10-13

**Authors:** Taylor C. Sutcliffe, Aimee N. Winter, Noelle C. Punessen, Daniel A. Linseman

**Affiliations:** 1 Department of Biological Sciences, University of Denver, Denver, CO 80208, USA; taylor.sutcliffe@gmail.com (T.C.S.); aimee.n.winter@gmail.com (A.N.W.); npunessen@gmail.com (N.C.P.); 2 Knoebel Institute for Healthy Aging, University of Denver, Denver, CO 80208, USA

**Keywords:** procyanidin B2, polyphenol, catechol, neurodegenerative diseases, antioxidant, free radicals, excitotoxicity, oxidative stress, nitrosative stress

## Abstract

The aberrant generation of oxygen and nitrogen free radicals can cause severe damage to key cellular components, resulting in cell apoptosis. Similarly, excitotoxicity leads to protease activation and mitochondrial dysfunction, which subsequently causes cell death. Each of these factors play critical roles in the neuronal cell death underlying various neurodegenerative diseases. Procyanidin B2 (PB2) is a naturally occurring polyphenolic compound found in high concentrations in cocoa, apples, and grapes. Here, we examine the neuroprotective effects of PB2 in primary cultures of rat cerebellar granule neurons (CGNs) exposed to various stressors. CGNs were pre-incubated with PB2 and then neuronal stress was induced as described below. Mitochondrial oxidative stress was triggered with HA14-1, an inhibitor of the pro-survival Bcl-2 protein which induces glutathione-sensitive apoptosis. Glutamate and glycine were used to induce excitotoxicity. Sodium nitroprusside, a nitric oxide generating compound, was used to induce nitrosative stress. We observed significant dose-dependent protection of CGNs with PB2 for all of the above insults, with the greatest neuroprotective effect being observed under conditions of nitrosative stress. Intriguingly, the neuroprotective effect of PB2 against nitric oxide was superoxide-dependent, as we have recently shown for other catechol antioxidants. Finally, we induced neuronal stress through the removal of depolarizing extracellular potassium and serum (5K conditions), which is a classical model of intrinsic apoptosis in CGNs. PB2 did not display any significant protection against 5K-induced apoptosis at any concentration tested. We conclude that PB2 offers neuronal protection principally as an antioxidant by scavenging reactive oxygen and nitrogen species instead of through modulation of pro-survival cell signaling pathways. These findings suggest that PB2 may be an effective neuroprotective agent for the treatment of neurodegenerative disorders.

## 1. Introduction

Neurodegeneration in critical areas of the brain and spinal cord leads to various diseases, such as Alzheimer’s disease (AD), Parkinson’s disease (PD), and amyotrophic lateral sclerosis (ALS). Oxidative and nitrosative stress have been implicated as possible causes of neuronal apoptosis and subsequent neurodegeneration that occurs in these diseases [1]. Oxidative and nitrosative stress result from the increased production of reactive oxygen species (ROS) and reactive nitrogen species (RNS), respectively. These conditions overwhelm cellular antioxidant defenses and can cause severe damage to key cellular components, ultimately resulting in cell death via apoptosis or necrosis [1,2,3].

Mitochondrial oxidative stress (MOS) has been strongly associated with the pathogenesis of various neurodegenerative diseases [1,4]. Because mitochondria continually perform oxidative phosphorylation at the electron transport chain to produce energy in the form of ATP, these organelles are a principal site of ROS formation. This process results in the leakage of electrons to molecular oxygen, forming the reactive species, superoxide, which can dismutate to form hydrogen peroxide (H_2_O_2_) and subsequently hydroxyl radicals [5]. These ROS can cause damage to mitochondrial components and initiate degradative processes by overwhelming the ability of the cell to enzymatically remove oxidatively damaged products [6]. This cellular disruption may ultimately extend to include dysfunction in energy metabolism, calcium homeostasis, stress response, and cell death pathways [7,8]. Further, glutathione (GSH), an essential endogenous antioxidant, has been found to be depleted in several neurodegenerative conditions [9,10,11]. This depletion prevents the cell from being able to scavenge reactive free radical species, which then target cellular components such as those found in mitochondria, resulting in more severe ROS production and increased oxidative stress. It is through this vicious cycle that the cell becomes overwhelmed and eventually undergoes apoptosis [4,12].

Nitrosative damage, caused primarily by nitric oxide radicals (NO^•^), has also been implicated in the pathologies of a variety of neurodegenerative disorders [2,13]. Specifically, *S*-nitrosylation, the covalent attachment of nitric oxide to a protein residue, has been shown to modify the function of a variety of proteins, resulting in neurodegenerative consequences [14,15,16,17]. These effects are due primarily to the *S*-nitrosylation of various proteins located in the cytosol or endoplasmic reticulum (ER), which innately function to maintain cell homeostasis and attenuate neurotoxic conditions [15]. In addition to its effect on proteins via *S*-nitrosylation of amino acid residues, nitric oxide can also damage DNA, causing extensive strand breakage, in addition to oxidation and subsequent depletion of endogenous antioxidants [18]. Furthermore, nitric oxide, an endogenous signaling molecule in neurotransmission, is also a potent vasodilator, which can enhance neuroinflammation, further perpetuating conditions of neurodegeneration [19].

Both oxidative and nitrosative stress have been found to be involved in specific neurodegenerative diseases. MOS, in particular, has been extensively studied and is strongly implicated as a key causative factor in the pathogenesis of PD, AD, and ALS. For instance, reactive metabolites of dopamine can initiate MOS, leading to the pathogenesis of PD [7,8,20]. The role of nitrosative damage and protein nitration has also been examined in relation to PD pathology [21]. Additionally, research has elucidated the connection between oxidative and nitrosative stress and AD [22,23]. Therefore, despite the different manifestations of these disorders, they share common oxidative and nitrosative stress components of their pathologies.

Despite the devastating effects of neurodegenerative diseases, few therapeutic options are currently available for afflicted individuals. For instance, the anti-glutamatergic agent, riluzole, is presently one of only two medications approved for the treatment of ALS, yet it is only able to prolong a patient’s lifespan for 2–3 months, while offering little alleviation of symptoms [24]. Similarly, few therapies exist for AD, as many drugs tested have been shown to be cost ineffective and minimally beneficial. Considerable evidence regarding the role of oxidative and nitrosative stress in the pathogenesis of these disorders has prompted research to examine antioxidant compounds that scavenge ROS and RNS as potential therapeutic options. In particular, research has begun to focus extensively on the naturally occurring group of antioxidants known as polyphenols or polyphenolic nutraceuticals.

Due to their powerful intrinsic antioxidant properties, polyphenols have become a major focus of study for treatment options for these disorders. Several compounds, including epigallocatechin 3-gallate (EGCG), found in green tea, and other polyphenols such as anthocyanins, have already been identified as effective ROS scavengers [25,26,27,28,29,30]. In fact, EGCG is effective in protecting against the loss of dopaminergic neurons located in the *substantia nigra pars compacta* through the inhibition of neuronal nitric oxide synthase (nNOS) in mice with MPTP-induced PD [31]. In addition to their ability to directly reduce oxidative stress, these compounds have also been shown to upregulate endogenous cellular defenses to indirectly protect neurons from degeneration [32]. The health benefits of polyphenols have also been shown to extend to ischemic injury, cancer, and cardiovascular disease prevention, among others [33,34,35]. Although the success of polyphenolic compounds in protecting against ROS is evident, their ability to alleviate the effects of nitrosative damage and excitotoxicity, other known causative factors of neurodegenerative diseases, is less clear.

**Figure 1:**
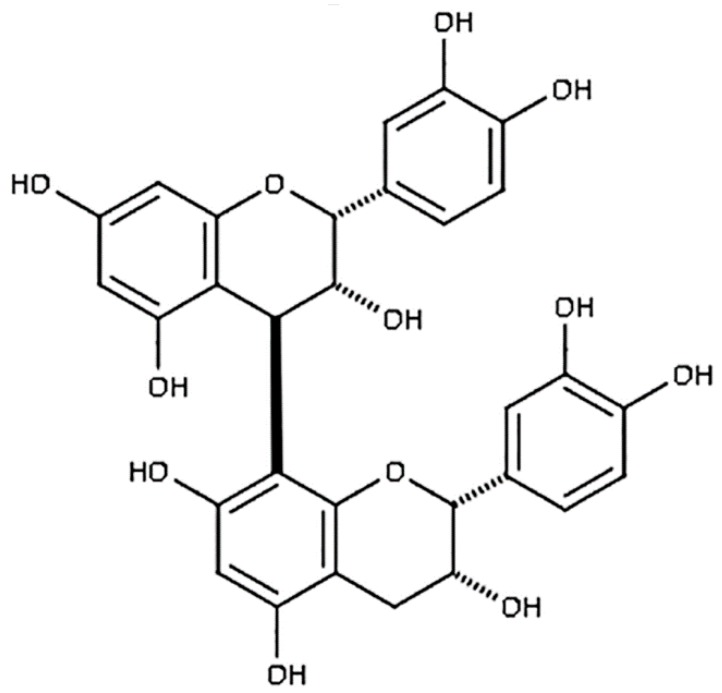
Chemical structure of PB2.

Procyanidin B2 (epicatechin-(4β-8)-epicatechin; PB2) is a potent antioxidant found naturally in high concentrations in cocoa, red grapes, apples, and cinnamon (Figure 1) [36,37]. It has been previously shown that PB2 protects PC12 cells from oxidative stress and subsequent apoptosis; however, it’s neuroprotective capabilities against nitrosative stress, excitotoxicity, and intrinsic apoptosis have not previously been examined [38,39]. Here, we investigate the neuroprotective properties of PB2, a member of the flavonoid family of nutraceuticals, against a variety of insults that simulate conditions similar to those observed in the context of neurodegeneration. Our results reveal a novel mechanism of protection from nitrosative stress in primary cerebellar granule neurons (CGNs) that is dependent on the ability of PB2 to generate superoxide; a finding which is similar to results we have recently published on another catechol antioxidant, the anthocyanin kuromanin [30]. Furthermore, we demonstrate that PB2 is an effective neuroprotective agent against a variety of neuronal stressors including MOS, nitrosative stress, and excitotoxicity.

## 2. Materials and Methods

### 2.1. Chemicals and Reagents

Procyanidin B2 (≥90% purity) was obtained from Extrasynthese (Genay, France). Glutamate, glycine, PEG-SOD, Hoechst stain, p-phenylenediamine, and monoclonal antibody against β-tubulin were purchased from Sigma-Aldrich (Saint Louis, MO, USA). HA14-1 (2-amino-6-bromo-α-cyano-3-(ethoxycarbonyl)-4H-1-benzopyran-4-acetic acid ethyl ester) and sodium nitroprusside (SNP) were obtained from Calbiochem (San Diego, CA, USA). FITC-conjugated secondary antibody was obtained from Jackson Immunoresearch Laboratories (West Grove, PA, USA). Nitric Oxide Assay kits (EMSNO) were obtained from Thermo Fisher Scientific (Rockford, IL, USA).

### 2.2. Cell Culture: Rat Cerebellar Granule Neurons

Primary cultures of cerebellar granule neurons (CGNs) were harvested weekly from both male and female seven day-old Sprague-Dawley rat pups as previously described [40]. CGNs were plated on poly-l-lysine coated 35-mm diameter six-well plates in Basal Medium Eagle’s at a density of approximately 4 × 10^6^ cells per well. Cell culture medium was supplemented with 25 mM potassium chloride, 10% fetal bovine serum, 2 mM l-glutamine, and penicillin/streptomycin (100 U/mL/100 μg/mL). Cells were incubated at 37 °C in 10% carbon dioxide for 24 h, after which 10 μL of 1 mM cytosine arabinoside (10 μM final concentration) were added to each well to prevent the growth of non-neuronal cells, such as astrocytes. This preparation yields CGNs of approximately 95% purity. Purity was visually confirmed prior to treatment, and impure cell cultures were not used in experimentation. CGNs were incubated under the same conditions for six or seven additional days before experimentation.

### 2.3. Protocol for Treatment with Procyanidin B2

In each experiment, cell culture medium was removed and replaced with 1 mL serum-free medium containing 25 mM KCl (25K-serum) immediately prior to pretreatment with PB2 to eliminate any possible neuroprotective effects of the serum. CGNs were first pretreated with PB2 at varying concentrations (10 μM to 80 μM) for six hours prior to exposure to one of several insults. HA14-1 (15 μM) or SNP (100 μM) were directly administered to the CGNs, and the cells were then incubated at 37 °C in 10% carbon dioxide for an additional 24 h. Similarly, in experiments in low potassium medium (i.e., 5K conditions), all cell culture medium was replaced with 25K-serum immediately prior to pretreatment with PB2. After this initial six-hour pretreatment incubation period, the medium in all experimental wells was replaced with 1 mL of 5K-serum medium, containing 5mM potassium, 2 mM L-glutamine, and penicillin/streptomycin (100 U/mL/100 μg/mL). PB2 was replaced in these wells during treatment with 5K-serum medium to maintain prior concentrations of PB2 for the entirety of the experiment. In experiments in which glutamate-induced excitotoxicity was examined, glutamate and its cofactor, glycine (100 μM/10 μM) were administered to cells following the 6-h pre-incubation period with PB2. CGNs were incubated in glutamate and glycine for 90 min at 37 °C in 10% carbon dioxide. All cell culture medium was then replaced with pure 25K-serum medium and PB2 was replaced to maintain prior concentrations of PB2 for the entirety of the experiment. Cells were incubated overnight at 37 °C in 10% carbon dioxide. All experiments included an untreated control and a control treated with only the insult for comparison in subsequent analyses. Each treatment was performed in duplicate or triplicate within every experiment.

### 2.4. Protocol for Treatment with PEG-SOD and SOD

Immediately prior to pretreatment, cell culture medium was replaced with 1 mL of 25K-serum medium. CGNs were pretreated with varying concentrations of PB2 (0 μM to 80 μM) and either SOD conjugated with polyethyleneglycol, (PEG-SOD; 30 U/mL) or unconjugated SOD (30 U/mL) for six hours prior to treatment with SNP. After a six-hour incubation period at 37 °C in 10% carbon dioxide, 100 μM SNP was then added to the appropriate wells, and plates were incubated for an additional 24 h. All experiments included an untreated control and a control treated with only SNP for comparison in subsequent analyses. All treatment conditions were duplicated without PEG-SOD or SOD for the same purpose. Each treatment was performed in duplicate or triplicate within every experiment.

### 2.5. Immunocytochemistry

After the 24-h treatment incubation period, all cell culture medium was removed, and CGNs were washed with 1× PBS. CGNs were fixed for 45 to 60 min in 4% paraformaldehyde, after which cells were washed again with 1× PBS. CGNs were incubated in blocking solution, consisting of 5% BSA in 0.2% Triton-X in PBS, for one hour at room temperature. Following blocking, cells were incubated with primary antibody to β-tubulin at 1:250 in 2% BSA and 0.2% Triton-X in PBS. Cells were next incubated in FITC-conjugated secondary antibody at a 1:250 dilution in 2% BSA in 0.2% Triton-X in PBS with Hoechst stain at 10 mg/mL for a minimum of one hour. CGNs were washed five times over the course of 30 min with 1× PBS. Cells were preserved in anti-quench solution containing PBS and *p*-phenylenediamine, and imaged using a Zeiss Axiovert-200M epifluorescence microscope (Carl Zeiss Microscopy, LLC, Thornwood, NY, USA).

### 2.6. Analysis of Apoptosis

Five representative images were taken of each well and analyzed to determine the percentage of apoptotic cells within each treatment condition. Nuclei were visualized using Hoechst fluorescence images, and nuclei were scored as either healthy or apoptotic. The percentage of nuclei displaying condensed and/or fragmented morphology, indicative of apoptosis, was then determined. Fragmented microtubule processes further confirmed apoptosis. In general, approximately 25–30 CGNs were quantified for apoptosis within each 40× field analyzed for a total of approximately 125–150 CGNs per well. Within a given experiment, each treatment was performed on duplicate or triplicate wells. As a result, each value represented in the individual bar graphs shown represents quantification of apoptosis in approximately 250–450 CGNs per treatment group.

### 2.7. MTT Viability Assay

As an alternative means of assessing cell death, some experiments were evaluated using an MTT viability assay. MTT (3-(4,5-dimethylthiazol-2-yl)-2,5-diphenyltetrazolium bromide) is a tetrazolium dye which is reduced by NAD(P)H-dependent cellular oxidoreductase enzymes, primarily within the mitochondria of viable cells, to yield an insoluble formazan derivative which can be solubilized and assayed colorimetrically as an indicator of cell viability. MTT data presented were obtained from duplicate wells per treatment shown for four independent experiments.

### 2.8. Nitric Oxide Assay

All nitric oxide assays were performed using Thermo Fisher Scientific’s Nitric Oxide Assay kit (Rockford, IL, USA). Cell-free samples were prepared in microcentrifuge tubes in 25K-serum medium containing SNP and varying concentrations of PB2. Controls were simultaneously prepared for comparison and contained only medium or only SNP and medium. Samples were sealed and incubated at 37 °C and 300 rpm overnight in an Eppendorf thermomixer. The nitric oxide concentration of each sample was assayed indirectly by measuring the total concentration of nitrate and nitrite in each sample using the Griess method per the manufacturer’s instructions using a BioTek PowerWave XS2 plate reader (BioTek Instruments, Inc., Winooski, VT, USA) and Gen5 software (BioTek Instruments, Inc., Winooski, VT, USA).

### 2.9. Data Analysis

Each experiment was performed a minimum of three times to ensure reproducibility. Within each experiment, each treatment was performed in duplicate or triplicate to decrease experimental uncertainty and error. Data were analyzed using a one-way ANOVA and a *post hoc* Tukey’s test. A *p* value < 0.05 was considered statistically significant.

## 3. Results

### 3.1. Procyanidin B2 Does Not Protect against 5K-Induced Apoptosis

**Figure 2:**
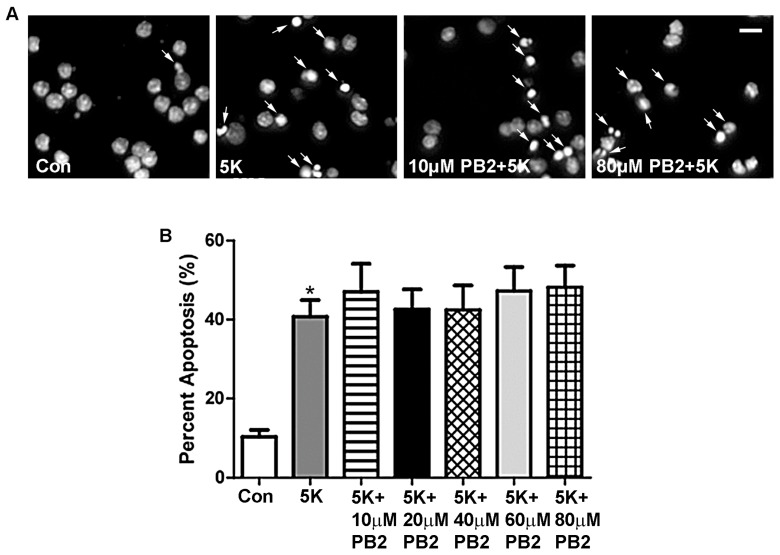
PB2 does not protect CGNs from 5K-induced apoptosis. (**A**) Representative images of CGNs treated for 24 h with varying concentrations of PB2 and 5K medium, 5K medium alone, and untreated control cells. Panels show decolorized Hoechst fluorescence to clearly indicate nuclear morphology. Scale bar = 10 μm. Arrows point to cells which were scored as apoptotic. (**B**) Quantitative analysis of percentage apoptosis of CGNs treated as in (**A**). Total cells and apoptotic cells were quantified and used to calculate percent apoptosis. Condensed or fragmented nuclei were considered to be apoptotic. Data are expressed as the mean ± the standard error of the mean (SEM), *n* = 3. Data were analyzed by one-way ANOVA with a *post hoc* Tukey’s test. * indicates *p* < 0.05 compared to untreated control. No significant differences were observed between the 5K control and 5K with the addition of any concentration of PB2.

We first examined the possible protective effects of PB2 against apoptosis induced by removing depolarizing potassium (5K). CGNs, which are harvested from early postnatal rats, require persistent calcium influx to mimic activity-dependent neuronal survival. However, in 5K conditions, cells are unable to maintain this calcium influx, which results in the induction of a mitochondrial (intrinsic) apoptotic death cascade [41,42]. Induction of intrinsic apoptosis was accomplished by incubating CGNs for 24 h in 5K medium, which lacks serum and depolarizing concentrations of potassium. PB2 did not significantly protect CGNs from 5K-induced apoptosis at any concentration tested (Figure 2). The nuclear morphology of CGNs was examined to determine the extent of neuronal apoptosis as a result of treatment with 5K medium. As shown in Figure 2A, cells treated with 5K medium alone or containing PB2 displayed fragmented and condensed nuclei (indicated by the arrows). This nuclear morphology indicates a large percentage of apoptosis, with little or no protective effects of PB2. These results are quantitatively analyzed in Figure 2B, which shows that treatment with 5K medium alone resulted in approximately 40% apoptosis, in comparison to 10% apoptosis for untreated controls. All cells treated with 5K medium, with or without PB2, were significantly more apoptotic than untreated control cells. Furthermore, there was no significant difference in levels of apoptosis observed in cells treated with PB2 in comparison to cells treated with 5K medium alone. These results indicate that PB2 does not protect CGNs from 5K medium-induced apoptosis (Figure 2B).

### 3.2. Procyanidin B2 Protects against Apoptosis Induced by the Bcl-2 Inhibitor HA14-1

**Figure 3:**
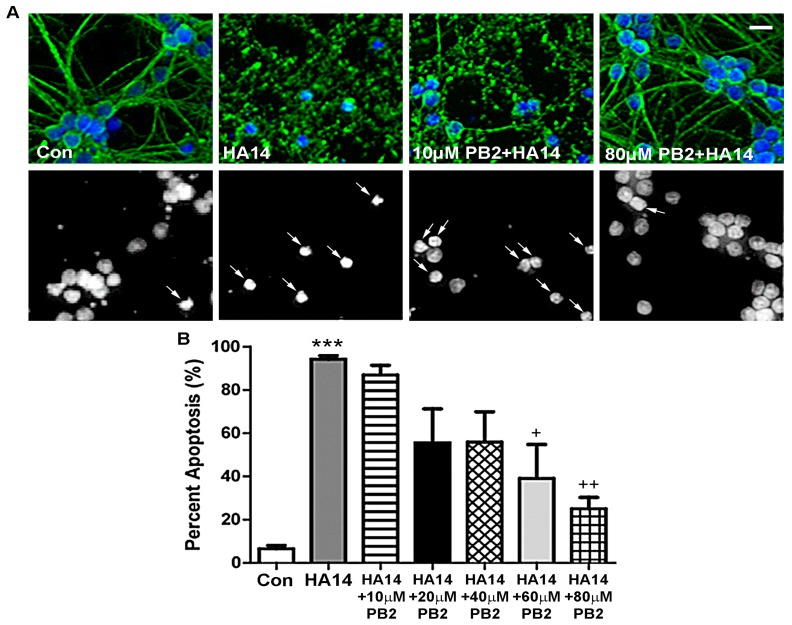
PB2 protects neurons from HA14-1-induced apoptosis in a dose-dependent manner. (**A**) CGNs were treated for 24 h with varying concentrations of PB2 and 15 μM HA14-1, 15 μM HA14-1 alone, and untreated control cells. Top panels show images of β-tubulin (green) and Hoechst stain (blue). Bottom panels show decolorized Hoechst fluorescence images of the same fields to better visualize nuclear morphology. Scale bar = 10 μm. Arrows point to cells which were scored as apoptotic. (**B**) Quantitative analysis of percentage apoptosis of CGNs in untreated controls and cells treated with both 15 μM HA14-1 and 0 μM to 80 μM PB2 for 24 h. Total cells and apoptotic cells were quantified and used to calculate percent apoptosis. Condensed or fragmented nuclei were considered to be apoptotic. Data are expressed as the mean ± SEM, *n* = 3. Data were analyzed by one-way ANOVA with a *post hoc* Tukey’s test. *** indicates *p* < 0.001 compared to untreated control. ++ indicates *p* < 0.01 and + indicates *p* < 0.05 compared to cells treated with only HA14-1.

HA14-1 is a small molecule that binds to the hydrophobic groove of the pro-survival protein, Bcl-2, effectively depleting levels of mitochondrial glutathione (GSH) and resulting in the activation of an intrinsic apoptotic pathway through induction of MOS [43,44,45]. As shown in Figure 3A, CGNs treated with HA14-1 alone displayed highly fragmented microtubule processes and condensed nuclei, which indicate a large percentage of apoptosis. Cells treated with HA14-1 and low concentrations of PB2 also displayed clear signs of apoptosis. However, higher concentrations of PB2 appeared to protect neurons from HA14-1, as indicated by the healthy morphology of nuclei and preserved microtubule processes (Figure 3A). These results are quantitatively analyzed in Figure 3B, which shows that PB2 significantly protects CGNs from HA14-1 in a dose-dependent manner. Treatment with HA14-1 alone induced roughly 95% apoptosis, which was significantly reduced by pretreatment with 60 μM PB2 and 80 μM PB2 to approximately 40% and 30% apoptosis, respectively (Figure 3B). These results indicate that PB2 is effective in protecting CGNs from HA14-1-induced MOS at concentrations ≥60 μM PB2.

### 3.3. Procyanidin B2 Protects CGNs from Glutamate-Induced Excitotoxicity

**Figure 4:**
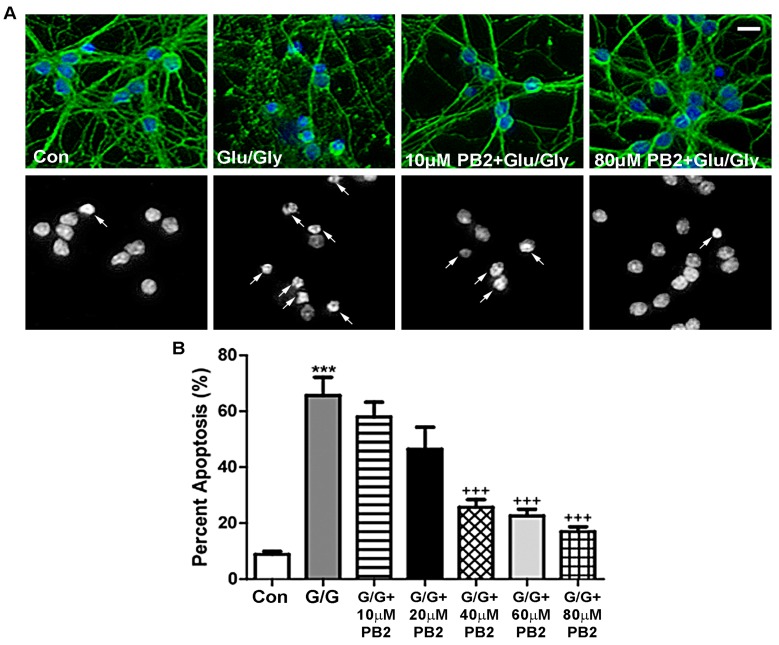
PB2 protects neurons from glutamate-induced excitotoxicity in a dose-dependent manner. (**A**) CGNs were treated with varying concentrations of PB2 and glutamate/glycine (Glu/Gly or G/G), glutamate/glycine alone, and untreated control cells. Top panels show staining for β-tubulin (green) and Hoechst nuclear dye (blue). Bottom panels show decolorized Hoechst fluorescence images of the same fields to better visualize nuclei. Scale bar = 10 μm. Arrows point to cells which were scored as apoptotic. (**B**) Quantitative analysis of percentage apoptosis of CGNs in untreated controls and cells treated with both glutamate and 0 μM to 80 μM PB2 for 24 h. Total cells and apoptotic cells were quantified and used to calculate percent apoptosis. Condensed or fragmented nuclei were considered to be apoptotic. Data are expressed as the mean ± SEM, *n* = 4. Data were analyzed by one-way ANOVA with a *post hoc* Tukey’s test. *** indicates *p* < 0.001 compared to untreated control. +++ indicates *p* < 0.001 compared to cells treated with only glutamate/glycine.

Excitotoxicity is a known causative factor in various neurodegenerative diseases that has both oxidative and nitrosative stress components [46]. Therefore, we next examined the neuroprotective potential of PB2 against glutamate-induced excitotoxicity, which induces apoptosis in CGNs via an NMDA receptor-dependent mechanism [47]. As shown in Figure 4A, cells treated with glutamate alone displayed slightly fragmented neuronal processes and significantly condensed and fragmented nuclei, indicating low cell viability. Conversely, CGNs pretreated with 80 μM PB2 before insult with glutamate displayed healthy nuclear morphology and preserved microtubule networks. These results are quantitatively analyzed in Figure 4B, which shows that PB2 protects CGNs from this insult in a dose-dependent manner. Alone, glutamate-induced excitotoxicity resulted in approximately 65% apoptosis (Figure 4B). Although PB2 did not significantly protect neurons at concentrations of 10 μM or 20 μM, we did observe significant protection at 40 μM, 60 μM, and 80 μM PB2, which reduced apoptosis to approximately 30%, 25%, and 20%, respectively (Figure 4B). These rates of apoptosis are approaching that observed in untreated control cells, which displayed roughly 10% apoptosis. These results indicate that PB2 is effective in protecting CGNs from glutamate-induced excitotoxicity at concentrations ≥40 μM PB2.

### 3.4. Evaulation of Procyanidin B2 Neuroprotective Effects by MTT Viability Assay

**Figure 5:**
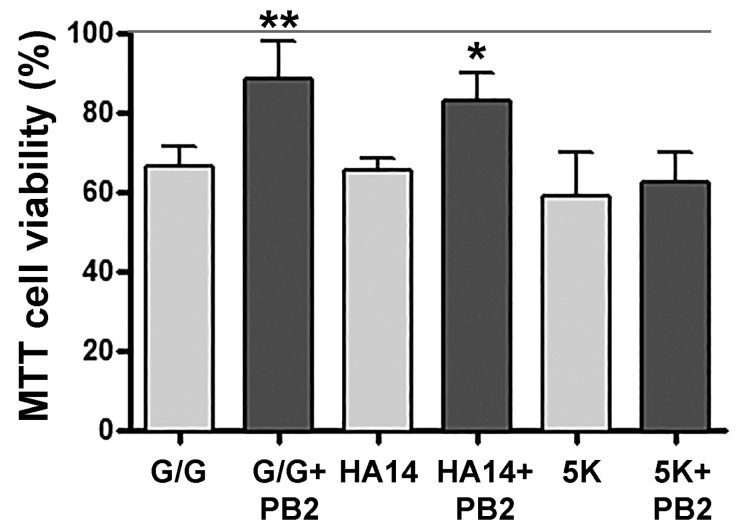
MTT viability assay to assess PB2 neuroprotection. CGNs were pre-incubated in 25K-serum control medium alone or containing 80 mM PB2, as described above, and then incubated for a further 24 h with either glutamate/glycine (G/G), HA14-1, or 5K apoptotic medium. Following incubation, an MTT viability assay was performed as described in Materials and Methods. The percentage viability is compared to cells incubated in control medium containing PB2 alone (100% line). The data represent the mean ± SEM viability for *n* = 4 independent experiments, each performed in duplicate. Data were analyzed by one-way ANOVA with a *post hoc* Tukey’s test. ** indicates *p* < 0.01 and * indicates *p* < 0.05 compared to each insult in the absence of PB2.

In order to use a more quantitative assay to evaluate the neuroprotective effects of PB2 in CGNs, we next measured cell viability using an MTT assay. As expected, CGN viability was significantly reduced by an average of 40% following treatment with either HA14-1, glutamate, or 5K apoptotic medium, as assessed by MTT assay (Figure 5). In excellent agreement with the results obtained using Hoechst staining and quantification of apoptotic nuclei (as described above), evaluation of CGN viability by MTT assay revealed that PB2 provided statistically significant protection from HA14-1 and glutamate, but not 5K apoptotic conditions (Figure 5).

### 3.5. Procyanidin B2 Protects CGNs against SNP-Induced Nitrosative Stress

**Figure 6:**
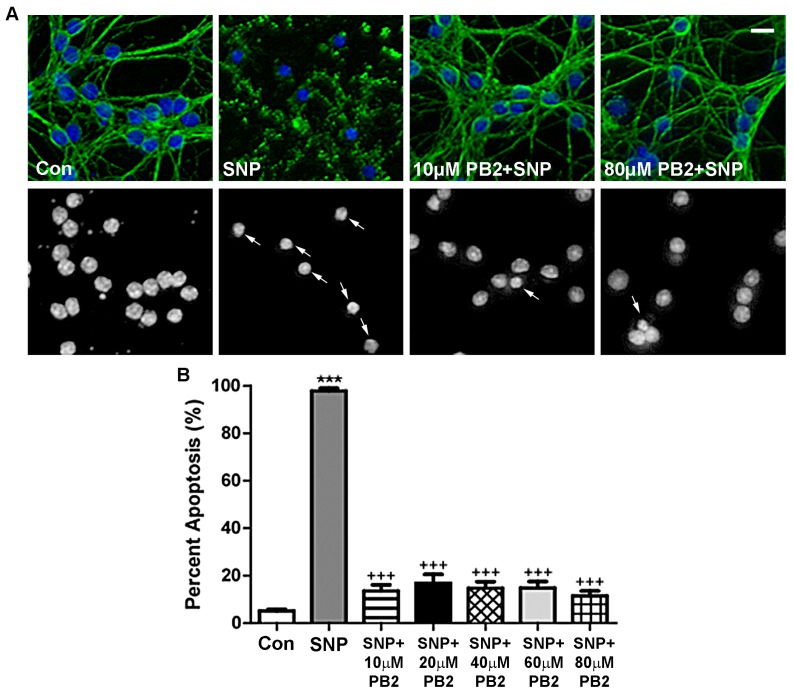
PB2 protects neurons from SNP induced nitrosative stress. (**A**) CGNs were treated for 24 h with varying concentrations of PB2 and SNP, SNP alone, and untreated control cells. Top panels show staining for β-tubulin (green) and Hoechst nuclear dye (blue). Bottom panels show black and white Hoechst fluorescence images of the same fields to better visualize nuclear morphology. Scale bar = 10 μm. Arrows point to cells which were scored as apoptotic. (**B**) Quantitative analysis of percentage apoptosis of CGNs in untreated controls and cells treated with both 100 μM SNP and 0 μM to 80 μM PB2 for 24 h. Total cells and apoptotic cells were quantified and used to calculate percent apoptosis. Condensed or fragmented nuclei were considered to be apoptotic. Data are expressed as the mean ± SEM, *n* = 4. Data were analyzed by one-way ANOVA with a *post hoc* Tukey’s test. *** indicates *p* < 0.001 compared to untreated control. +++ indicates *p* < 0.001 compared to cells treated with only SNP.

Nitrosative damage, in addition to oxidative stress and excitotoxicity, plays a key role in neurodegeneration and neuroinflammation. Nitric oxide, an endogenous signaling molecule, is also a potent vasodilator [48]. However, under neurodegenerative conditions, nitric oxide can also contribute to MOS by increasing the prevalence of ROS and RNS in the cell. As illustrated in Figure 6A, CGNs treated with the nitric oxide donor, SNP, displayed highly fragmented neuronal processes and condensed nuclei, which, together, indicate a high degree of apoptosis. In contrast, cells pretreated with PB2 prior to the addition of SNP displayed healthy nuclear morphology and preserved microtubule networks that are comparable to untreated control cells (Figure 6A). These results are quantitatively analyzed in Figure 6B, which shows that PB2 almost completely protects CGNs from this insult, even at a concentration as low as 10 μM PB2. Alone, SNP-induced nitrosative damage caused almost 100% apoptosis (Figure 6B). Conversely, the rates of apoptosis in cells treated with both SNP and PB2 are comparable to the untreated control cells, indicating the efficacy of PB2 in preventing neuronal damage caused by nitric oxide (Figure 6B). These results indicate that PB2 has a high propensity to protect CGNs from SNP-induced nitrosative damage at concentrations ≥10 μM PB2.

### 3.6. Procyanidin B2 Reduces Nitric Oxide Concentration in a Cell-Free System

**Figure 7:**
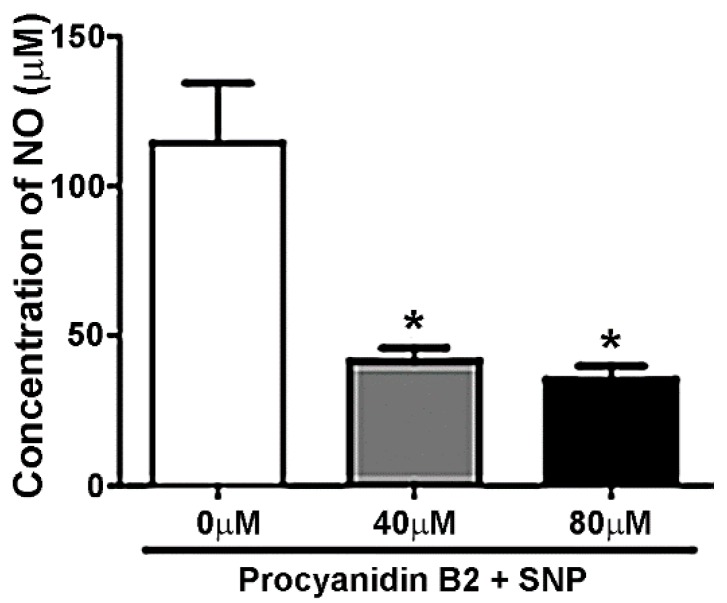
PB2 Reduces Nitric Oxide Concentration Produced by SNP in a Cell-Free System. Quantitative analysis of nitric oxide generation by SNP in the presence of PB2. Data are expressed as the mean ± SEM, *n* = 3. * indicates *p* < 0.05 in comparison to samples containing SNP alone. See Materials and Methods section for a detailed explanation of the experimental protocol.

Due to the high efficacy of PB2 in protecting CGNs from SNP-induced toxicity, we next sought to determine the final nitric oxide concentration in cell-free samples containing medium alone, SNP alone, and PB2 and SNP using a nitric oxide assay. Because nitric oxide is an unstable free radical, it can react rapidly in solution to generate both nitrate and nitrite, which were measured using the Griess method to indirectly determine the concentration of nitric oxide generated by SNP in solution [49]. As shown in Figure 7, PB2 at concentrations of 40 μM and 80 μM, significantly reduced the concentration of nitric oxide which was spontaneously produced in cell culture medium by SNP. These data indicate that PB2 has the innate capacity to scavenge nitric oxide in solution.

### 3.7. PEG-SOD Prevents PB2 from Protecting CGNs against SNP-Induced Toxicity

Previous research has shown that antioxidants, such as polyphenols which possess catechol moieties, also have pro-oxidant characteristics [50]. Indeed, PB2 has been shown to have both anti- and pro-oxidant effects in the human leukemia cell line HL-60 treated with H_2_O_2_ [36]. Moreover, our previous work has shown that the neuroprotective effects of a related compound, cyanidin-3-*O*-glucoside (kuromanin), against SNP-induced nitric oxide toxicity are dependent on the ability of this compound to generate superoxide radicals [30]. Superoxide generation appeared to be dependent on the catechol structure of this compound, a common feature shared between kuromanin and PB2. Given this prior research and the ability of PB2 to reduce concentrations of nitric oxide in a cell-free system, we hypothesized that PB2 might defend CGNs from nitric oxide in a similar manner.

Therefore, we next treated CGNs with SNP alone or following pretreatment with PB2, in the absence or presence of either unconjugated SOD or PEG-SOD, to further elucidate this potential mechanism. SOD catalyzes the dismutation of superoxide into H_2_O_2_, a species that is no longer able to react with nitric oxide to form peroxynitrite (ONOO^−^). However, unconjugated SOD is not able to pass through the phospholipid bilayer and therefore, would not be able to scavenge superoxide produced by PB2 intracellularly. Conversely, SOD that is covalently linked to polyethylene glycol (PEG-SOD) is able to permeate the cell membrane [51]. Therefore, we hypothesized that, if PB2 is generating superoxide anions as a way to scavenge nitric oxide radicals intracellularly, then PEG-SOD, but not SOD, should reverse the neuroprotective effects of PB2 in the presence of SNP.

**Figure 8:**
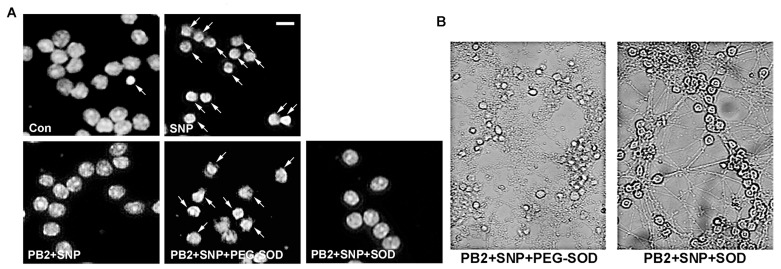

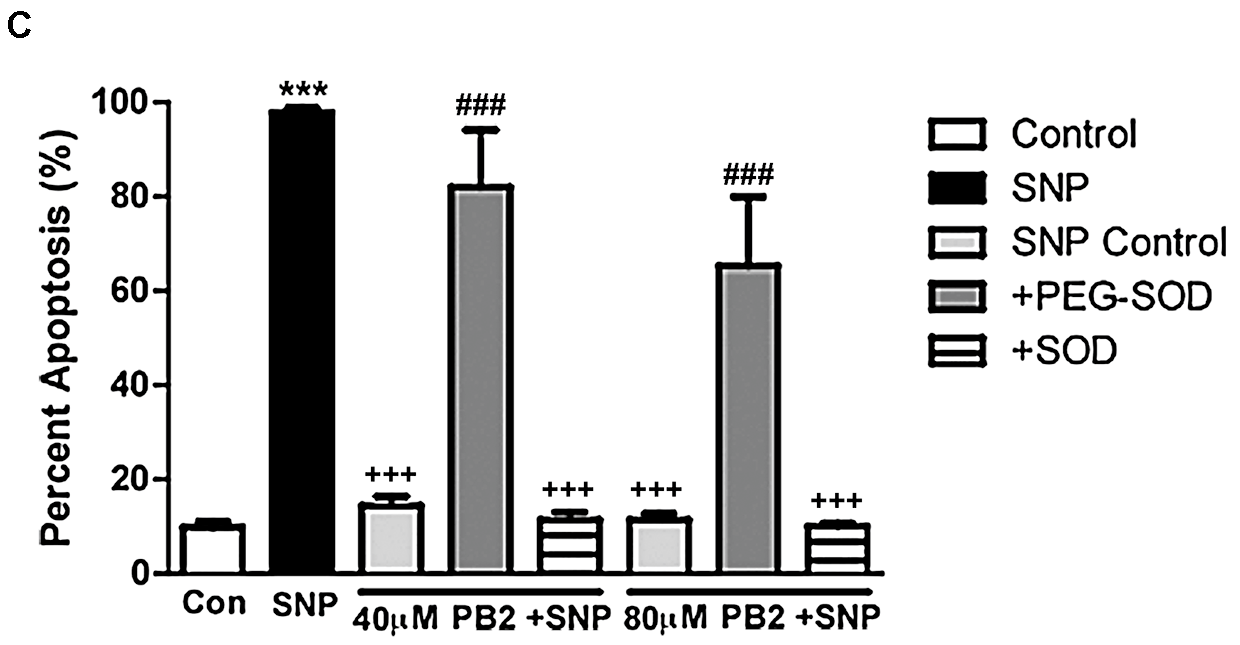
PEG-SOD, but not unconjugated SOD, prevents neuronal protection by PB2 in the presence of SNP. (**A**) CGNs were treated for 24 h with 100 μM SNP alone or following pretreatment with 80 μM PB2 in the absence or presence of either 30 U/mL PEG-SOD or 30 U/mL unconjugated SOD, and untreated control cells. The images show black and white Hoechst staining of neuronal nuclei. Scale bar = 10 μm. Arrows point to cells which were scored as apoptotic. (**B**) Bright field images of CGNs treated with SNP + PB2 and either unconjugated or PEG-conjugated SOD to illustrate neuronal process morphology. (**C**) Quantitative analysis of percentage apoptosis of CGNs in untreated controls and cells treated with 100 μM SNP alone or in combination with 40 μM or 80 μM PB2 with or without 30 U/mL PEG-SOD or unconjugated SOD for 24 h. Total cells and apoptotic cells were quantified and used to calculate percent apoptosis. Condensed or fragmented nuclei were considered to be apoptotic. Data are expressed as the mean ± SEM, *n* = 3. Data were analyzed by one-way ANOVA with a *post hoc* Tukey’s test. *** indicates *p* < 0.001 compared to untreated control. +++ indicates *p* < 0.001 compared to cells treated with only SNP. ### indicates *p* < 0.001 compared to cells treated with SNP and PB2.

As shown in Figure 8, our results confirm our hypothesis that PEG-SOD, but not SOD, was able to reverse the neuroprotective effects of PB2 against SNP in CGNs. As described above (see Figure 6), cells pretreated with PB2 prior to the addition of SNP were completely protected, as indicated by the healthy nuclear morphology and intact processes of the cells (Figure 8A,B). Alone, both PEG-SOD and SOD were non-toxic to cells at the concentrations used (30 U/mL; data not shown). The neuroprotective effects of PB2 against SNP toxicity were maintained in cells co-treated with SOD, which is impermeable to the plasma membrane (Figure 8A,B). However, cells co-treated with PB2 and the cell-permeable PEG-SOD were no longer protected from SNP toxicity and a clear reversal of the neuroprotective effect was observed (Figure 8A,B). These results are quantitatively analyzed in Figure 8C, which shows that PB2 is effective at protecting CGNs from SNP when PB2 is administered either alone or in combination with unconjugated SOD. However, neuronal protection is reversed when PB2 is administered in combination with PEG-SOD (Figure 8C). These data suggest that PB2 likely generates superoxide, which can combine intracellularly with nitric oxide to produce ONOO^−^, thus attenuating the toxic effects of nitric oxide and preventing neuronal cell death.

## 4. Discussion and Conclusions

PB2 is a polyphenolic compound which possesses two catechol moieties (Figure 1). Here, we show that PB2 demonstrates significant neuroprotective effects in primary cultures of CGNs against both oxidative and nitrosative stressors, as well as against glutamate-induced excitotoxicity. In all three of these neurotoxic conditions, PB2 preserved the integrity of the microtubule network of CGNs and prevented the induction of apoptotic nuclear morphology, in stark contrast to cells treated with the insult alone. However, PB2 did not appear to influence 5K-induced apoptosis, which is a classical model of intrinsic (mitochondrial) apoptosis in CGNs. Therefore, these results suggest that PB2 might be an effective therapeutic option for the treatment of some neurodegenerative diseases for which underlying oxidative and nitrosative stress are significant contributors. In contrast, PB2 does not appear to be a strong modulator of pro- or anti-apoptotic signaling pathways in CGNs.

We initially found that PB2 was effective at preserving CGNs in the presence of MOS induced by HA14-1, an inhibitor of Bcl-2 and an inducer of GSH-sensitive apoptosis in CGNs [43,44,45]. Because MOS is a well-known and extensively studied component of several neurodegenerative diseases, it is beneficial that PB2 is protective against this inducer of apoptosis. For instance, Mecocci and colleagues showed an increase in oxidative damage to mitochondrial DNA within the cerebral cortex and cerebellum of AD patients as compared to age-matched controls [22]. Many other studies have also shown a link between MOS and PD [8,52,53]. Specifically, both mitochondrial dysfunction and mitochondrial oxidative damage, resulting in the inactivation of complex I, were found in the substantia nigra and frontal cortex of PD patients [52]. Further research has also established a link between MOS and organelle dysfunction and ALS pathogenesis [54,55,56]. However, because the pathogenesis of neurodegenerative diseases is caused by factors in addition to oxidative stress, it is crucial to assess the ability of PB2 to offer neuroprotection from a variety of other relevant insults to further assess its viability as a therapeutic agent.

Excitotoxicity, another known causative agent of neurodegeneration, was also evaluated in the context of PB2 neuroprotection. PB2 was found to protect against glutamate-induced excitotoxicity at concentrations lower than those required to protect CGNs from MOS induced by HA14-1. This result is in agreement with a previous study by Shimada and colleagues which showed that PB2, in addition to a variety of other phenolic compounds including epicatechin, catechin, procyanidin B1, hyperin, and caffeic acid, protected against glutamate-induced death in CGNs [57]. These findings further reflect the promising efficacy of PB2 as a therapeutic agent, as excitotoxicity has been implicated in several neurodegenerative diseases. In particular, excitotoxicity appears to play a considerable role in the pathogenesis of ALS, as clearly evidenced by the mechanism through which Riluzole, currently one of only two approved medications for the treatment of ALS, acts to block glutamate signaling [58]. Research has also directly shown that glutamate levels are increased in the cerebrospinal fluid of ALS patients [59]. Several studies have shown depleted clearance or reuptake of extracellular glutamate from the synapse, which could, in part, lead to the pathology of ALS [60,61]. Glutamate-induced excitotoxicity has also been implicated in AD and PD and is currently being pursued as a novel target of neuroprotective agents in these diseases [62,63].

Despite the ability of PB2 to protect against oxidative stress and excitotoxicity, PB2 shows an even higher propensity to protect CGNs from nitric oxide. Of particular note, PB2 completely protected CGNs from SNP-induced toxicity at concentrations as low as 10 μM, approximately 4–6× lower than those required to protect against oxidative stress and excitotoxicity. Under physiologically healthy conditions, nitric oxide is produced endogenously to act as a signaling molecule, resulting in the *S*-nitrosylation of proteins, as well as acting as a vasodilator [48]. However, this process can become dysregulated in neurodegenerative conditions when nitric oxide is aberrantly generated, causing the excessive *S*-nitrosylation and aggregation of certain affected proteins, as well as compromising various pro-survival pathways in the cell [14,18]. Thus, we were keenly interested in elucidating the mechanism by which PB2 is so effective at protecting neurons against nitric oxide-induced toxicity.

Polyphenolic compounds, found in various fruits and vegetables, are widely recognized for their antioxidant properties. It is generally accepted that polyphenols act as antioxidants by either directly scavenging reactive species or indirectly affecting endogenous cellular defenses to remove free radicals [64]. In particular, these compounds can quench free radical species, as well as chelate transition metals, which exemplify their ability to directly act on ROS or RNS [35]. Furthermore, polyphenols can also activate enzymes, such as glutathione reductase and glutathione peroxidase, which in turn, upregulate the production of the endogenous antioxidant compound, GSH. These antioxidant qualities of polyphenols have driven the assessment of many compounds as potential treatments for disorders, such as neurodegenerative diseases.

In addition to the apparent antioxidant characteristics of polyphenols, recent research has also garnered support for the pro-oxidant qualities of these compounds. In particular, several studies have found that polyphenolic compounds are able to suppress the growth of tumor cells by inducing apoptosis, largely through their pro-oxidant effects [35,65]. However, it is important to note that studies show that polyphenols preferentially target cancerous cells for apoptosis, whereas these compounds promote protection in normal tissue [65]. Specifically, it is believed that these pro-oxidant effects can induce endogenous antioxidant systems in healthy tissue while simultaneously inducing apoptosis in tumors via the same mechanism [65].

To further elucidate the mechanistic function of PB2, we treated CGNs with PEG-SOD or unconjugated SOD. SOD dismutates superoxide into H_2_O_2_, which is not able to react with nitric oxide to form ONOO^−^. However, SOD is not able to permeate the lipid bilayer, but PEG-SOD, which is conjugated to polyethylene glycol, is cell-permeable. Therefore, if PB2 is offering its neuroprotective effects against nitric oxide through an intracellular mechanism, then PEG-SOD, but not SOD, should reverse neuroprotection by PB2 in CGNs treated with the nitric oxide donor, SNP. Accordingly, we found that CGNs treated with PEG-SOD were no longer protected from SNP-induced toxicity in the presence of PB2. Conversely, the neuroprotective effects of PB2 were unaffected in cells treated with unconjugated SOD. This finding brings up an interesting paradox: how does PB2 depend on the generation of superoxide radical to protect CGNs from nitric oxide toxicity?

**Scheme 1:**
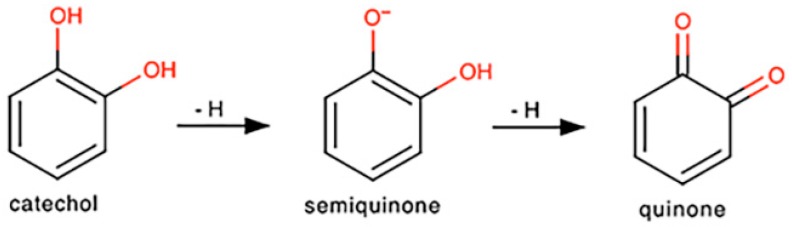
Proposed mechanism of PB2 auto-oxidation. Catechol is first oxidized to become semiquinone, which is again oxidized, becoming a quinone [66,67]. One superoxide anion is generated for each auto-oxidation step.

As discussed in Chichirau et al., many polyphenols share a common functional group, a catechol, and it is through this structural unit that these compounds execute their pro-oxidant capabilities [66]. As shown in Scheme 1, catechols can spontaneously undergo two auto-oxidation reactions, ultimately resulting in the formation of a quinone [66,67]. However, for each oxidation, the compound generates a molecule of superoxide. This anion can then react with nitric oxide to generate ONOO^−^, a more stable species [19]. Although ONOO^−^ is able to interact with lipids, DNA, and proteins, which can result in overwhelming oxidative injury at high concentrations, Bolaños and colleagues have shown that, at low concentrations, ONOO^−^ can modify protein residues, thus activating various anti-apoptotic pathways [19]. ONOO^−^ has also been linked to the stimulation of glucose metabolism and GSH regeneration, further protecting the cell from apoptosis [19]. Furthermore, ONOO^−^ has been found to mitigate the neurotoxicity elicited by excess nitric oxide by activating processes such as DNA repair or glucose-6-phosphate dehydrogenase [19,68]. Therefore, we hypothesize that PB2, through its two catechol moieties, generates superoxide via auto-oxidation, which then reacts with nitric oxide to form ONOO^−^, which is less toxic to CGNs and results in neuroprotection from nitric oxide.

Our previous work has demonstrated that kuromanin, another catechol-containing polyphenol of the anthocyanin family, is capable of producing superoxide in addition to scavenging nitric oxide in a cell-free system [30]. Given these observations, there is a strong likelihood that PB2 acts in an identical manner, although we did not explicitly test the potential of PB2 to generate superoxide in cell culture medium. Moreover, it would be worthwhile to compare the protective effects of PB2 to similar compounds, such as kuromanin, in order to determine which compound shows the greatest neuroprotective and therapeutic potential for treating diseases for which nitric oxide toxicity is a significant factor. Indeed, given the similar results between these two studies, it is possible that the neuroprotective effects of polyphenols against nitric oxide work through a general catechol mechanism that is not specific to a particular compound. Furthermore, because PB2 has two catechol groups in its chemical structure, this might suggest superior neuroprotection against nitrosative damage over compounds that possess only one of these functional groups (e.g., kuromanin). It is important to note, however, that procyanidins can often be large structures, so it might be more difficult for these compounds to cross biological membranes, resulting in diminished bioavailability in vivo [69]. Nevertheless, studies have shown that PB2 is stable in the stomach environment and is readily absorbed at the intestine, suggesting that this compound can be ingested orally while maintaining its integrity [70]. Research has also found that PB2 and other similar compounds are seen in human plasma within 30 min of ingestion, with concentrations peaking at 2 h, and the absorption of PB2 appears to be greater than that of related compounds, such as procyanidin B3 and procyanidin C2 [71,72]. Importantly, a study by Serra and colleagues found that procyanidin metabolites were able to cross the blood-brain barrier in rats that ingested procyanidin-rich cocoa cream, though it remains to be seen if PB2 itself can be found in brain tissue [73]. In this context, a recent study showed that intra-gastric administration of PB2 following middle cerebral artery occlusion in rats attenuated neurological deficits and blood brain barrier disruption in this model of cerebral ischemia [10]. However, it should be noted that these authors did not assess levels of PB2 within brain tissue in this study.

It is also important to note that procyanidin-rich extracts, which include PB2, do not appear to cause negative effects at the organismal level. Specifically, Yamakoshi and colleagues examined acute and subchronic oral toxicity in Fischer 344 rats that were orally administered procyanidin-rich extracts from grape seeds [74]. However, there were no observed adverse effects of this treatment, even at relatively high concentrations (1410 mg/kg body weight/day in males; 1501 mg/kg body weight/day in females) [74]. Together, these findings support the use of procyanidin-rich extracts in vivo as potential therapeutic agents for neurodegeneration.

In sum, this research highlights the neuroprotective effects of PB2 in the presence of oxidative and nitrosative stressors, as well as glutamate-induced excitotoxicity. These data suggest that PB2 might, in fact, offer a therapeutic option for individuals currently suffering from neurodegenerative diseases, such as AD, PD, and ALS. While PB2 does not protect from all neurotoxic insults, PB2 might be effective in conjunction with a variety of other neuroprotective agents. Specifically, if PB2 is paired with other neuroprotective compounds that are more efficient in modulating pro-survival or pro-apoptotic cell signaling pathways, then this combinatorial medication could be effective in alleviating the suffering of those afflicted with these debilitating disorders.

## Abbreviations

AD	Alzheimer’s disease
ALS	amyotrophic lateral sclerosis
CGNs	cerebellar granule neurons
GSH	glutathione
H_2_O_2_	hydrogen peroxide
HA14-1	2-amino-6-bromo-α-cyano-3-(ethoxycarbonyl)-4H-1-benzopyran-4-acetic acid ethyl ester
iNOS	inducible nitric oxide synthase
MOS	mitochondrial oxidative stress; nNOS, neuronal nitric oxide synthase
NO^•^	nitric oxide
O_2_^•−^	superoxide anion
^•^OH	hydroxyl radical
ONOO^−^	peroxynitrite
PB2	procyanidin B2
PBS	phosphate buffered saline
PD	Parkinson’s disease
PEG-SOD	polyethylene glycol conjugated superoxide dismutase
RNS	reactive nitrogen species
ROS	reactive oxygen species
SNP	sodium nitroprusside
SOD	superoxide dismutase
